# Largest single-institution series of robotic pneumonectomy

**DOI:** 10.3389/fsurg.2026.1841553

**Published:** 2026-06-24

**Authors:** Ammar Asban, Nikolaos Pachos, Caroline A. Snyder, Isabella Ferraro, Stephanie Chang, Costas Bizekis, Robert J. Cerfolio, Michael D. Zervos

**Affiliations:** Department of Cardiothoracic Surgery, NYU Langone Health, New York, NY, United States

**Keywords:** lung cancer, minimally invasive surgery, pneumonectomy, robotic pneumonectomy, robotic surgery

## Abstract

**Background:**

Minimally invasive pneumonectomy via robotic or video-assisted platforms is not yet widely adopted. Rates of conversion-to-thoracotomy and 90-day mortality are reported at 30%–50% and 12%, respectively. We describe our experience with the largest single-institution consecutive series of robotic pneumonectomy.

**Methods:**

This is a retrospective review of prospective database of a consecutive (non-selected) series of patients who underwent robotic pneumonectomy from January 2019 to December 2024 at our institution.

**Results:**

There were 21 patients (67% male) with median age 70 (range 15–79), DLCO 76% (range 48%–106%), and FEV1 76% (range 44%–126%). Indication for pneumonectomy was cancer in 17 patients (81%), of which 15 (88%) had a primary lung cancer. Four patients (19%) had destroyed lung from infection, and six (29%) underwent completion pneumonectomy. Thirteen (76%) of the 17 cancer patients had neoadjuvant therapy, including chemotherapy (11 patients), immunotherapy (10 patients), and radiation therapy (6 patients). A robotic approach was selected for all patients, with planned conversion-to-open in two patients (9%). There were no intraoperative transfusions. Median chest tube duration was one day (range 1–4). Median length of stay was five days (range 1–25). Thirty-day readmission was 19% (4 patients). Thirty-day morbidity was 19%, including atrial fibrillation in two patients, hemothorax in one patient, and chylothorax in one patient. Thirty-day mortality was 0% and 90-day mortality was 5% (one patient).

**Conclusion:**

Pneumonectomy can be performed safely and effectively robotically even in high-risk patients with previous ipsilateral thoracotomy and/or neoadjuvant chemo-, immuno-, or radio-therapy. We demonstrate that a robotic approach enables minimal unplanned conversion-to-thoracotomy, no blood transfusions and excellent short-term outcomes. Our pre-, intra- and post-operative strategies are shared.

## 1 Introduction

Currently, more pulmonary lobectomies are performed robotically than open or video-assisted in the United States (1). A minimally invasive approach has demonstrated equal if not superior outcomes and survival rates when compared with video-assisted thoracoscopic surgery (VATS) and open approaches (2). Moreover, robotic surgery has demonstrated superior intraoperative and postoperative outcomes including postoperative pain, length of stay (LOS), and conversion-to-open rates (2).

However, robotic pneumonectomy has not yet been fully adopted by thoracic surgeons across the globe, largely due to the risk of stapling the main pulmonary artery and bleeding. This was the same argument against robotic lobectomy when we first started it in 2008 (3). In addition, almost all patients that undergo pneumonectomy have had extensive hilar disease, T3 or T4 tumors, neo-adjuvant induction chemo-immunotherapy and/or radiotherapy, or destroyed lungs from infection such as mycobacterium avium intracellular (MAI). We rarely offer pneumonectomy for most patients with multilevel bulky N2 disease, as these patients must have had relatively long-term survival without development of metastatic disease to even consider the option. We present our experience with robotic pneumonectomy and describe our surgical approach, operative techniques, and short-term outcomes.

## 2 Methods

### Data source and patient selection

This is a retrospective review of prospective database of a consecutive (non-selected) series of patients who underwent robotic pneumonectomy from January 2019 to December 2024 at our institution. Those who had an unplanned conversion from a robotic approach to an open thoracotomy were included in this study and defined as have an “unplanned conversion to thoracotomy.” Patients who were planned to have a thoracotomy after an initial robotic platform assessment because of tumor size or other reasons were defined as a “planned conversion to thoracotomy.” All age groups were included. There were no exclusion criteria. This study was approved by the NYU Langone Health Institutional Review Board (i24- 00491). A total of three thoracic surgeons performed the procedures included in this series.

### Surgical indications

Patients were evaluated in a multidisciplinary thoracic oncology conference including thoracic surgeons, medical oncologists, radiation oncologists, pulmonologists, and radiologists. Patients with locally advanced NSCLC who initially presented with advanced-stage disease, including stage IIIC or oligometastatic stage IV disease, were considered for surgical resection only after completion of induction systemic therapy and demonstration of substantial treatment response on restaging imaging. In patients with biopsy-proven N3 disease, surgery was considered only in the setting of complete metabolic resolution of involved nodal stations on post-treatment PET-CT imaging and absence of disease progression. Patients undergoing pneumonectomy for benign disease had chronically destroyed lungs secondary to infection and were selected for surgery after failure of prolonged medical management or development of recurrent complications.

### Outcomes measurement and statistical analysis

Demographic variables reported include age, gender, race, BMI, ECOG score (0–5), preoperative DLCO and FEV1 in percentage, and smoking history (current, former, or never smoked). Preoperative comorbidities reported include hypertension, diabetes, coronary artery disease, chronic kidney disease, and lung disease including history of COPD/emphysema or pulmonary hypertension. We also collected data on neoadjuvant chemotherapy, immunotherapy, or radiation therapy. Additionally, we obtained data on indication for pneumonectomy, preoperative lung cancer staging, operative (OR) time, the requirement for intraoperative blood transfusion, intraoperative complications, and estimated blood loss (EBL). Post-operative outcomes measured were length of stay, intensive care unit admission, time until chest tube removal, discharge home with chest tube, postoperative complications (atrial fibrillation, surgical site infection, pneumothorax, hemothorax, or chylothorax requiring chest tube placement), 30-day readmission rate and reason for readmission, and 30- and 90-day mortality rate. Finally, we reported the final pathological stage and resection status. Continuous variables were summarized as median and range. All statistical analyses were performed using Stata SE 14.2.

### Patient positioning, port placement and intraoperative strategy

All procedures were performed using the da Vinci Xi Surgical System (Intuitive Surgical, Sunnyvale, CA, USA). The patient is positioned in the lateral decubitus position. We use a right-sided double-lumen endotracheal tube for a left pneumonotomy and a left-sided tube for right pneumonectomy. We insert the robotic ports over the ninth rib. The ports are placed as follows if in the right chest: robotic arm 1 (8-mm port) is 4 cm lateral to the spinous process of the vertebral body; robotic arm 2 (8 mm) is 8 cm medial to robotic arm 4; the camera port (8-mm camera) is 8 cm medial to robotic arm 3; and robotic arm 4 (12 mm) is anteriorly above the diaphragm. The assistant port is triangulated behind the most anterior robotic port and the camera port, as inferiorly as possible without disrupting the diaphragm. We use a zero-degree camera. The camera first then the assistant port is insufflated with carbon dioxide to depress the diaphragm, compress the lung, and reduce bleeding (4). Our strategy for pulmonary artery control follows the principles we previously described (5). Intraoperatively, arterial blood gas analysis is routinely performed while the patient is on 100% FiO₂ to assess adequacy of single-lung ventilation and gas exchange. A temporary pulmonary artery test clamp is performed prior to definitive division to evaluate potential hemodynamic compromise. Transesophageal echocardiography is used to monitor cardiac function during test clamping as well as before and after pneumonectomy.

## 3 Results

### Patient demographics and clinical characteristics

From January 2019 to December 2025, there were 21 patients who underwent pneumonectomy at our institution. All patients were offered a robotic approach. The study cohort included 14 (67%) men, 12 (57%) white race/ethnicity, and median values of age 70 (range 15–79), BMI 24 (range 16–38), DLCO 76% (range 48%–106%), and FEV1 76% (range 44%–126%). Most patients had an ECOG score of zero (ten patients, 47%), and most were former smokers (13 patients, 62%). Sixteen patients hard (76%) had left pneumonectomy. Table 1 illustrates the patient demographics and clinical characteristics. Seventeen (81%) patients underwent pneumonectomy for cancer, and 15 (88%) had primary lung cancer. Table 2 lists the surgical indication for pneumonectomy. Stage IIIA disease was most common (six patients, 35%). Among lung cancer patients, thirteen patients received neo-adjuvant therapy of which 11 (52%) received neoadjuvant chemotherapy, ten (48%) received neoadjuvant immunotherapy, and six (29%) received neoadjuvant radiation therapy. Surgical pathology demonstrated non-small cell lung cancer in 15 patients (71%).

**Table 1:** Patient demographics and clinical characteristics.

Characteristic	Cohort (*n*=21)
Age (years) – median (range)	70 (15–79)
Male sex – no. (%)	14 (66.67)
Race – no. (%)	
White	12 (57.14)
Black	1 (4.76)
Other	8 (38.10)
BMI – median (range)	24.44 (16.33–37.72)
ECOG – no. (%)	
0	10 (47.62)
1	9 (42.82)
2	2 (9.52)
FEV1 – median (range)	76 (44–126)
DLCO – median (range)	76 (48–106)
Smoking status at time of surgery – no. (%)	
Never smoker	6 (28.57)
Former smoker	13 (61.90)
Current smoker	2 (9.52)
Pack-year history – median (range)	23 (0–100)
HTN – no. (%)	6 (28.57)
DM – no. (%)	4 (19.05)
CAD – no. (%)	6 (28.57)
Lung disease – no. (%)	10 (47.62)
COPD	7 (33.33)
Emphysema	7 (33.33)
Pulmonary HTN	2 (9.52)
Other	4 (19.05)
Preoperative anticoagulation – no. (%)	9 (42.82)
Clopidogrel	2 (9.52)
Direct oral anticoagulant (DOAC)	1 (4.76)
Other	6 (28.57)
Neoadjuvant therapy – no. (%)	
Chemotherapy	11 (52.38)
Immunotherapy	10 (47.62)
Radiation therapy	6 (28.57)
Preoperative final pathologic stage – NSCLC 8th edition – no. (%)	
IA3	1 (5.88)
IIB	4 (23.53)
IIIA	6 (35.29)
IIIC	2 (11.76)
IVA	3 (17.65)
IVB	1 (5.88)

**Table 2:** Surgical indications for pneumonectomy.

Indication	Cohort (*n* = 21)
Primary lung cancer	15 (70%)
Metastatic pulmonary disease	2 (10%)
Granulomatous pulmonary disease	2 (10%)
Bronchiectasis/organized pneumonia	2 (10%)

### Operative approach and intraoperative outcomes

**Figure 1:**
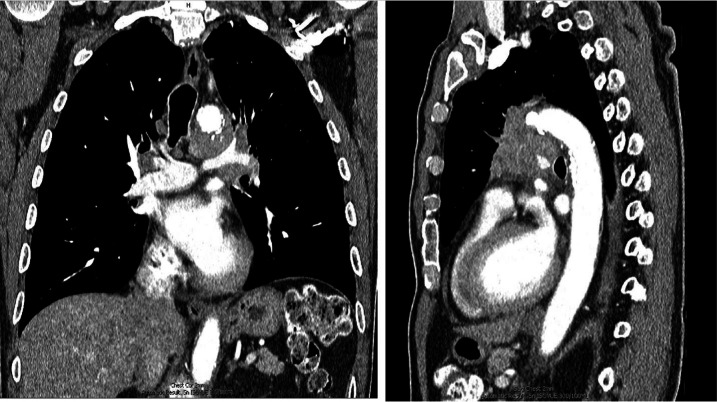
Central tumor encasing the left pulmonary artery and invading the aortic arch successfully resected via robotic pneumonectomy.

All 21 patients underwent robotic pneumonectomy with a planned conversion-to-open in two patients (9%). Conversion was planned due to tumor involving the superior pulmonary vein in one patient, and tumor invasion into the esophagus and aorta in the second patient. The median operative time was 190 min (range 146–354). There were no intraoperative transfusions or any major intraoperative complications. The median blood loss was 50 mL (range 20–250). All patients had intrapericardial dissection of the main pulmonary artery. Pulmonary artery stapling was performed using a SureForm 45, white load stapler (Intuitive Surgical, Sunnyvale, CA, USA). Figure 1 illustrates a central tumor encasing the pulmonary artery and invading the aortic arch.

### Postoperative outcomes

Four (19%) patients went to the ICU for overnight monitoring; these cases occurred early in our experience with robotic pneumonectomy. The median LOS was five days (range 1–25). However, LOS was one day in the most recent two patients, and LOS decreased to 1–5 days in the most recent five patients, with most patients discharged on postoperative day two. Most patients had their chest tube removed on postoperative day one (13 patients, 62%). The most common final pathological stages were stage IIB and IIIA disease (four patients, 27% each). Thirty-day morbidity occurred in four (19%) patients (one hemothorax, one chylothorax, two atrial fibrillation). Four (19%) patients were readmitted, although none for postoperative complications. Readmission reason was rehabilitation placement in two patients, vomiting in one patient, and orthostatic hypotension in one patient. Thirty-day mortality was 0% and 90-day mortality was 5% (one patient). This patient passed away at an outside institution, not from complication related to pneumonectomy. All perioperative and postoperative outcomes are reported in Table 3.

**Table 3:** Perioperative outcomes.

Outcome	Cohort (*n* = 21)
Mass type – no. (%)	
Adenocarcinoma	7 (33.33)
Squamous cell carcinoma	7 (33.33)
Other (including non-neoplastic)	7 (33.33)
Robotic approach – no. (%)	21 (100)
Conversion to open – no. (%)	2 (9.52)
Operation time (minutes) – median (range)	190 (146–354)
Intraoperative transfusion – no. (%)	0 (0)
Intraoperative complication – no. (%)	0 (0)
Estimated blood loss (mL) – median (range)	50 (20–250)
Postoperative intensive care unit stay – no. (%)	4 (19.05)
Length of stay (days) – median (range)	5 (1–25)
Day chest tube removed (days) – median (range)	1 (1–4)
Patients discharged with chest tube – no. (%)	0 (0)
Postoperative complications – no. (%)	
Pneumothorax	0 (0)
Hemothorax	1 (4.76)
Myocardial infarction	0 (0)
Atrial fibrillation	2 (9.52)
Surgical site infection	**0 (0)**
N1 Nodes, stations and med # and [range]	**3 stations, 5 nodes (3–36)**
N2 Nodes, stations and med # and [range]	**5 stations, 16 nodes (5–51)**
Postoperative Final Pathologic Stage – NSCLC 8th Edition – no. (%)	
IA2	1 (6.67)
IB	1 (6.67)
IIB	4 (26.67)
IIIA	4 (26.67)
IIIC	1 (6.67)
IVA	3 (20.00)
IVB	1 (6.67)
R0 resection – no. (%)	21 (100)
Readmission – no. (%)	4 (19.05)
30-day mortality – no. (%)	0 (0)
90-day mortality – no. (%)	1 (4.76)

Bold values indicates N1 Nodes and N2 Nodes, stations and median # [range].

### Recurrence and survival

**Figure 2:**
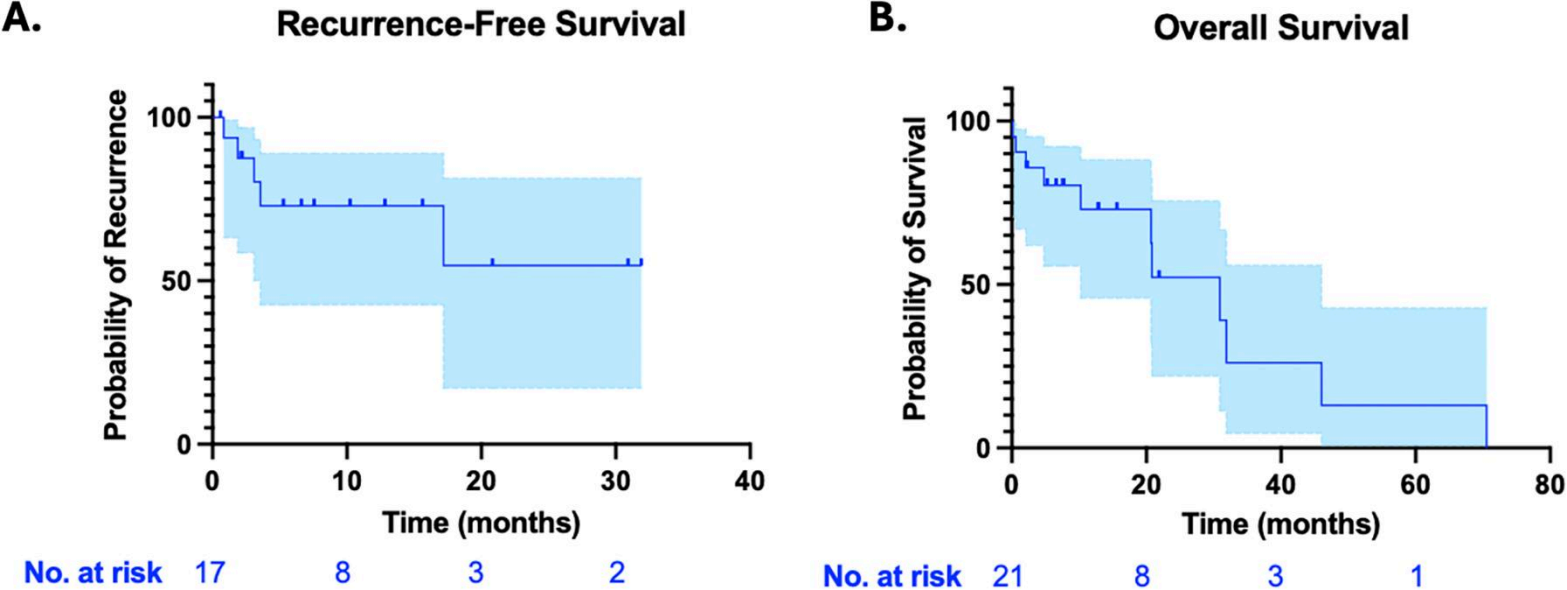
Kaplan–meier survival analysis. **(A)** Recurrence free survival in patients who underwent pneumonectomy for lung malignancy (*n* = 17). **(B)** Overall survival in all patients (*n* = 21). Shaded areas represent 95% confidence intervals.

Follow-up was complete for all patients (median 10 months, range 1–70 months). Five patients (24%) had recurrent disease, with recurrence diagnosed at a median of three months after surgery. Among patients with one-year follow-up data, there was 60% survival. Kaplan–Meier curves for recurrence and overall survival are shown in Figure 2.

## 4 Discussion

In this study, which is the largest reported single-institution series of robotic pneumonectomy to date, we demonstrate that even for complex and large invasive tumors in patients that have undergone neoadjuvant therapy, a robotic approach is safe and effective with outstanding intra-operative metrics, 0% 30-day mortality, 5% 90-day mortality, and 60% one-year survival in patients with extended follow-up.

In a National Cancer Database study from 2010 to 2020 by Trope et al., the authors investigated factors associated with successful minimally invasive pneumonectomy. They reported that only 20% of patients who underwent pneumonectomy were offered a minimally invasive approach to start, and in these patients, conversion-to-thoracotomy was 31% (6). Higher conversion rates were associated with lower volume centers. Similarly, Shah et al. reviewed the National Cancer Database for patients who underwent pneumonectomy from 2015 to 2020. (2) This study found that only 8% of the cohort underwent robotic pneumonectomy. Additionally, even among those who have adopted a robotic approach, the conversion-to-open rate was high, reaching 30%–50% (2, 7). Mack et al. also reviewed the National Cancer Database for robotic pneumonectomy from 2014 to 2018, identifying 4,838 patients total, of which only 269 patients (5.7%) were completed robotically (8). The authors also found that a robotic approach did not affect the number of lymph nodes harvested or R0 resection, but there was a shorter LOS compared to VATS or open approaches. There was no difference in 5-year overall survival rate between open, VATS, and robotic approaches (50%, 49%, and 55%, respectively) (8). Additionally, there was no difference in 30-day and 90-day survival rate between robotic (94% and 92%), open (93% and 89%), and VATS (93% and 89%) (8). Finally, Patton et al. reported a single-institution pneumonectomy experience for 13 lung cancer patients (7). In that report, a robotic approach was successful in eight patients (53%), with a conversion rate of 38%. Interestingly, the authors found that more lymph nodes were harvested robotically, with less operative time and blood loss (7). However, there was no difference between the robotic and conversion-to-thoracotomy group of patients when comparing LOS, ICU stay, or chest tube removal day. Major complications after robotic pneumonectomy included bronchopleural fistula and vocal cord paralysis in one patient each, and atrial fibrillation in one patient (7). Historically, pneumonectomy carries significant morbidity and mortality regardless of approach, with 90-day mortality rates of 7%–12% reported in large national and multi-institutional series (9–11). This risk is further increased after neoadjuvant therapy, with a meta-analysis reporting 90-day mortality of 12% overall and up to 20% for right-sided pneumonectomy (12). In this context, our 90-day mortality of 5% compares favorably, despite the high proportion of patients who received neoadjuvant chemo-immunotherapy or radiation.

In our series, we offered all patients a robotic platform and our conversion rate was 9%, with both conversions planned and a 0% unplanned conversion rate. We believe these outcomes are directly related to our processes which include: (1) obtaining main pulmonary artery control inside the pericardium (which we often do for difficult lobectomy after neo-adjuvant therapy) and placing a vessel loop that is double-wrapped around the main pulmonary artery in order to reduce its diameter to enable easier stapler application to control it (as shown in Supplementary Video S1); and (2) cutting the bronchus first if it is adherent to the pulmonary artery and using a handheld stapler on the bronchus after it is often first cut with a bi-polar. In our study, there were two planned conversions. In one patient, the tumor was involving the superior pulmonary vein and invading into the left atrium, and conversion was planned to resect the tumor and oversew the left atrium. In the second patient, conversion was planned because of the tumor size of 17 cm and the fact that it was invading the esophagus and onto the aorta. Pneumonectomy was performed because the lung was destroyed and infected, and the patient had recurrent significant hemoptysis that had failed all medical therapies over several months.

This study has several limitations including the retrospective and descriptive nature of the study, the small sample size that precludes performing more complex statistical analysis and the absence of a comparative VATS or open pneumonectomy cohort, as pneumonectomy is routinely performed robotically at our institution. The follow-up is short and limits long-term survival conclusions. Additionally, this series represents a highly selected cohort that would rarely be considered for surgery at most centers, particularly those patients who initially presented with stage IIIC and IV disease. The strength of this study is that it represents the largest single-institution series of robotic pneumonectomy reported in the literature, including many variables and reporting some of the best outcomes to date. Most importantly, and the main import for writing this paper is that we believe our experience is scalable to other centers via process improvements. We have found from proctoring and from courses that we are involved in that these specific processes and steps outlined above are teachable. In our experience, we have found that the main hurdle is not the experience or talent of the surgeon, but rather the culture of innovation and acceptance in their institution or hospital system. There is often little tolerance for change and innovation and thus the surgeon too often does not feel safe to even try a minimally invasive pneumonectomy. Once the non-data driven dogma that “pneumonectomy has to be performed via a thoracotomy” is debunked, more patients can enjoy the benefits of a minimally invasive pneumonectomy.

## Data Availability

The raw data supporting the conclusions of this article will be made available by the authors, without undue reservation.

## Supplementary material

Supplementary Video 1 This videos demonstrates placement of a vessel loop that is double wrapped around the main pulmonary artery for proximal control.
